# Redefining Pulmonary Arterial Hypertension Treatment: Sotatercept's FDA Priority Review

**DOI:** 10.7759/cureus.66253

**Published:** 2024-08-06

**Authors:** Harendra Kumar, Arkadeep Dhali, Jyotirmoy Biswas, Gopal Krishna Dhali

**Affiliations:** 1 General Medicine, Dow University of Health Sciences, Karachi, PAK; 2 Internal Medicine, Sheffield Teaching Hospitals National Health Service (NHS) Foundation Trust, Sheffield, GBR; 3 General Medicine, College of Medicine and Sagor Dutta Hospital, Kolkata, IND; 4 Gastroenterology, School of Digestive and Liver Diseases, Institute of Postgraduate Medical Education and Research, Kolkata, IND

**Keywords:** outcome, therapy, activin, pulmonary hypertension, sotatercept

## Abstract

A biologics license application (BLA) for sotatercept, a therapeutic agent targeting activin receptor signaling implicated in pulmonary arterial hypertension (PAH), has been granted priority review status by the FDA. This advancement underscores the critical need for novel pharmacological interventions for this rare and severe condition, potentially transforming the therapeutic landscape of PAH.

## Editorial

A biologics license application (BLA) for sotatercept, an inhibitor of activin signaling used in treating pulmonary arterial hypertension (PAH), has received a priority review from the FDA [1]. This development signals the pressing demand for innovative medications addressing this rare and life-threatening disease, potentially reshaping PAH treatment.

PAH involves the stiffening and constriction of lung blood vessels, leading to elevated pulmonary artery pressure and impacting the heart's right side [1,2]. Symptoms include breathlessness, fatigue, chest tightness, and fainting. PAH often leads to right-sided heart failure and increased mortality [2,3].

Approximately 75,000 Americans suffer from PAH, which typically carries a poor prognosis, with a median life expectancy of five to seven years post-diagnosis [4]. Current PAH treatments focus on dilating lung capillaries and easing heart strain but do not target the root cause - the abnormal formation and restructuring of pulmonary vascular cells [1,2].

Sotatercept works in the pulmonary arteries by blocking activins and growth differentiation factors. These proteins help cells grow and stop them from dying. By balancing cell growth and death again, sotatercept may be able to stop or reverse the changes in the blood vessels that cause PAH [1].

In clinical trials, sotatercept has shown significant and clinically meaningful improvements in several parameters of PAH severity and quality of life. In the phase 3 STELLAR research, adding sotatercept to background medicine increased the six-minute walk distance (6MWD), a measure of exercise capacity, by 40.8 m compared to the placebo after 24 weeks [1]. Sotatercept also helped with pulmonary vascular resistance, N-terminal pro-B-type natriuretic peptide level, WHO functional class, time to death or clinical deterioration, French risk score, and three PAH-SYMPACT questionnaire categories [1,2].

These results were published in the New England Journal of Medicine and presented earlier this year at the American College of Cardiology Scientific Session [1]. According to a recent study presented at the European Respiratory Society International Congress, sotatercept improved the size and function of the right heart as well as blood flow in the lungs of PAH patients, as measured by tests that measure pressure and oxygen levels in the heart and blood vessels. According to these results, sotatercept may improve the heart and lungs function in patients with PAH, potentially improving survival and quality of life [2]. According to a preliminary analysis of the phase 3 SOTERIA follow-up study, sotatercept was safe and effective after one year [1]. Sotatercept could be a game changer for PAH patients, who have few options and face a high risk of illness and death. Sotatercept has been prioritized for study by the FDA as a new and substantial drug that has the potential to improve safety or effectiveness in treating, diagnosing, and preventing PAH [1-3]. A priority review means that the FDA will try to decide on the BLA within six months rather than the usual 10 months. Patients with PAH who need treatment today may find it easier to get sotatercept.

Sotatercept is a novel activin signaling inhibitor that targets the vascular proliferation underlying PAH [5]. In the STELLAR trial (Table 1), sotatercept improved PAH patients' ability to exercise, their blood flow, and their functional class on top of their regular treatment [1]. Based on these results, the FDA granted sotatercept a priority review for its BLA in September 2023. This designation recognizes the unmet medical need and the potential clinical benefit of sotatercept for PAH patients. Sotatercept represents a promising new therapeutic option for this life-threatening disease.

**Table 1:** Clinical trials of sotatercept: efficacy and outcomes

Trial	Phase	Population	Intervention	Comparator	Primary Outcome	Result
STELLAR [1]	Phase 3	Adults with pulmonary arterial hypertension (PAH) (World Health Organization (WHO) functional class II or III)	Sotatercept (0.3-0.7 mg/kg) subcutaneously every three weeks	Placebo subcutaneously every three weeks	Change from baseline at week 24 in six-minute walk distance (6MWD)	Sotatercept improved 6MWD by 40.8 m compared to placebo (P < 0.001)
SOTERIA [2]	Phase 3 (open-label extension)	Adults with PAH who completed the STELLAR trial	Sotatercept (0.3-0.7 mg/kg) subcutaneously every three weeks	N/A	Safety and durability of the efficacy of sotatercept for up to five years	Sotatercept was well tolerated and maintained its efficacy improvements after one year of therapy
ACE-011 [3]	Phase 2	Adults with anemia of myeloproliferative neoplasm (MPN)-associated myelofibrosis (MF)	Sotatercept (0.75-1 mg/kg) subcutaneously every three weeks	N/A	Anemia response, defined as achievement of transfusion independence or increase in hemoglobin (Hgb) level from baseline of ≥1.5 g/dl sustained for ≥12 weeks	Sotatercept induced anemia response in 8 of 27 (30%) evaluable patients

Conclusion

The FDA's priority evaluation of sotatercept underlines its potential to significantly improve PAH therapy. Sotatercept is a possible option for improving outcomes and quality of life in patients suffering from the incapacitating disease PAH by treating both symptoms and fundamental causes.

## References

[1] Hoeper MM, Badesch DB, Ghofrani HA (2023). Phase 3 trial of sotatercept for treatment of pulmonary arterial hypertension. N Engl J Med.

[2] Preston IR, Badesch D, Ghofrani HA (2023). Late breaking abstract—a long-term follow-up (LTFU) study of sotatercept for pulmonary arterial hypertension (PAH). Eur Respir J.

[3] Raftopoulos H, Laadem A, Hesketh PJ (2016). Sotatercept (ACE-011) for the treatment of chemotherapy-induced anemia in patients with metastatic breast cancer or advanced or metastatic solid tumors treated with platinum-based chemotherapeutic regimens: results from two phase 2 studies. Support Care Cancer.

[4] McLaughlin V, Alsumali A, Liu R (2024). Population health model predicting the long-term impact of sotatercept on morbidity and mortality in patients with pulmonary arterial hypertension (PAH). Adv Ther.

[5] Souza R, Badesch DB, Ghofrani HA (2023). Effects of sotatercept on haemodynamics and right heart function: analysis of the STELLAR trial. Eur Respir J.

